# Microstructural Evolution and Mechanical Properties of LPBF Ti-6Al-4V with Different Process Parameters

**DOI:** 10.3390/ma19061049

**Published:** 2026-03-10

**Authors:** Yuxin Shuai, Jie Liu, Jing Zhu, Zhichao Huang, Wenhao Zha, Yi Yang, Ruifeng Zhang, Kai Zhang

**Affiliations:** 1 School of Materials and Chemistry, University of Shanghai for Science and Technology, Shanghai 200093, China; s1389060222@163.com (Y.S.); jzzdqy1@hotmail.com (J.Z.); 2 School of Energy and Environment, Southeast University, Nanjing 210096, China; liujie1998221@hotmail.com; 3 School of Iron and Steel, Soochow University, Suzhou 215002, China; 20245249037@stu.suda.edu.cn (Z.H.); 20245249016@stu.suda.edu.cn (W.Z.); 4 School of Materials Science and Engineering, Central South University, Changsha 410083, China; 5 Monash Centre for Additive Manufacturing (MCAM), Monash University, Melbourne, VIC 3168, Australia; 6 Department of Materials Science and Engineering, Monash University, Clayton, VIC 3800, Australia

**Keywords:** laser powder bed fusion, Ti-6Al-4V, process parameters, texture, variant selection, process–microstructure relationship

## Abstract

Although processing windows have been widely reported for LPBF Ti-6Al-4V, the distinct roles of laser power, scanning speed, and hatch distance remain unclear beyond VED-based comparisons. In this work, the distinct effects of laser power, scanning speed, and hatch distance on the microstructural evolution and mechanical response of laser powder bed fusion (LPBF) Ti-6Al-4V (Ti64) are investigated within a stable processing window with comparisons among different parameter combinations at a comparable VED. A total of 56 processing conditions were designed, and microstructure/texture and properties were characterized by OM/SEM, EBSD, microhardness (HV0.5), and hole-drilling residual stress measurements. Within the selected processing window, prior-β grain morphology, α’ martensite thickness, texture, microhardness, and residual stress exhibit distinct sensitivities to different processing parameters. Specifically, lower scanning speeds and smaller hatch distances promote more continuous <001>β epitaxial growth, whereas higher scanning speeds or larger hatch distances produce fragmented prior-β grains. The α’ lath thickness shows the strongest dependence on scanning speed with a secondary influence from hatch distance, while laser power mainly provides an overall thermal modulation. Furthermore, the macroscopic α (0002) texture is mainly governed by the β solidification texture, with α-variant selection playing a secondary, amplifying role. In addition, microhardness correlates with α’ martensite thickness following a Hall–Petch equation. The peak residual stress is more sensitive to scanning speed, while bulk residual stress varies more significantly with hatch distance. These findings demonstrate that process parameters, in addition to VED, can guide microstructural control and mechanical optimization in LPBF Ti64 alloy.

## 1. Introduction

Laser powder bed fusion (LPBF) is a digital model-based additive manufacturing technology that selectively melts and rapidly solidifies metal powders layer by layer to fabricate complex-shaped components directly [1,2,3,4]. This process offers high geometric freedom and dimensional accuracy while significantly reducing material waste and manufacturing time [5,6,7]. Ti-6Al-4V (Ti64), a typical dual-phase (α + β) titanium alloy, is widely used owing to its excellent mechanical performance and strong corrosion resistance [8,9]. LPBF Ti64 alloy exhibits higher yield and tensile strengths compared to conventionally processed Ti64 alloys, making it widely applicable across various industries [10]. Microstructure features, including prior-β grain, α laths, and crystallographic texture, strongly govern the yield/tensile strength and elongation of LPBF Ti64 [1,9,11,12,13,14,15,16,17]. Refining the prior-β grains improves both tensile strength and elongation [18]. Similarly, refined martensitic laths confer high hardness and yield strength, while simultaneously resulting in reduced elongation [9,10,16,19,20,21]. In addition, a pronounced crystallographic texture promotes mechanical anisotropy, where tensile strength increases along the preferred orientation, whereas ductility decreases because slip activity becomes more constrained [15,22]. Beyond tensile/yield strength and ductility, process-tailored microstructures and residual stresses are broadly recognized to affect service reliability in aggressive environments (e.g., oxide/deposit evolution and crack initiation), as demonstrated across multiple alloy systems under high-temperature aqueous conditions [23,24,25,26]. Moreover, LPBF-built Ti64 has been reported to show better corrosion resistance in acidic and oxidizing media than cast Ti64 [27].

LPBF process parameters (e.g., laser power, scanning speed, and hatch distance) influence the microstructural evolution of LPBF titanium alloys and consequently enable the tailoring of their mechanical properties [10,28,29]. These process parameters determine the heat input and thermal history of the molten pool [16,30,31,32,33]. The cooling rate of the molten pool is the critical factor determining microstructure features like α lath thickness [16,19,32,33]. Increasing the scanning speed and decreasing the laser power can promote faster cooling, thereby leading to the formation of fine acicular α’ martensite via a diffusionless β → α’ martensitic transformation upon cooling [16,20,34,35]. The morphology of prior-β grains is also governed by the process-dependent heat input and cooling rate, where a stable thermal gradient favors columnar grain growth, whereas rapid cooling promotes the formation of equiaxed grains [1,29]. Moreover, the β → α transformation in α-β titanium alloys occurs during the LPBF process, where only a subset of the 12 Burgers variants usually forms in each prior-β grain. This α-variant selection can markedly intensify the transformation texture [36,37]. Cooling strongly affects this process: slower cooling and more diffusional β → α growth favor a limitation of low-energy variants and thus strengthen the texture, whereas higher cooling rates promote finer lamellae, greater variant diversity and consequently weaker, more randomly oriented textures [36,38,39,40]. Consistent with this, slower cooling rates generally enhance texture intensity, while rapid cooling is associated with weaker and more random textures [41].

Volumetric energy density (VED) is widely used to quantify the nominal energy input per unit volume in LPBF, because it is straightforward to calculate and provides a convenient metric for comparing process conditions and correlating with defects and mechanical properties [42,43,44,45]. Nevertheless, recent studies have shown that VED has limited predictive capability for microstructural evolution and mechanical performance [46,47]. This limitation arises because VED reduces laser power, scanning speed, and hatch distance to a single scalar value, which cannot fully represent the complex interactions among these parameters and the resulting thermal histories. As a result, substantial variations in microstructure and mechanical properties can still occur under similar VED values [46,48]. For instance, in LPBF Ti64 fabricated at a comparable VED, a higher scanning speed combined with a smaller hatch distance promotes finer grains, whereas a lower scanning speed and a larger hatch distance tend to produce columnar grain [47,49]. Similarly, a 14% difference in ultimate tensile strength has been reported between two Al-12Si samples produced with identical VED but different laser power–scanning speed combinations [50]. Collectively, these observations indicate that while VED reflects the overall energy input, it cannot capture parameter-specific thermal histories that ultimately govern detailed microstructure formation.

To this end, a more parameter-resolved understanding beyond VED is required to clarify how individual LPBF variables drive microstructural evolution and mechanical performance. It is generally accepted that LPBF process parameters influence microstructure and mechanical properties. However, how specific laser power, scanning speed, and hatch distance govern microstructural evolution and thereby affect mechanical performance remains insufficiently understood. In many cases, specimens fabricated under similar or even optimized processing conditions still exhibit markedly different prior-β grain morphologies, α/α’ features, and mechanical responses. This inconsistency indicates that the underlying process–structure link is not yet clearly defined, particularly with respect to the distinct roles of individual parameters.

Despite recent efforts on parameter windows and microstructure control, the influence of laser power, scanning speed, and hatch distance on microstructure and mechanical performance in LPBF Ti-6Al-4V remain unclear beyond VED-based frameworks. In this study, a parameter combination approach was employed to systematically elucidate the process–structure–property relationships in LPBF Ti64 alloy. A total of 56 process parameter sets were designed to cover a wide range of process windows, enabling quantitative analysis of the effects of laser power, scanning speed, and hatch distance. First, the effects of laser power, scanning speed, and hatch distance on prior-β grain morphology, α’ lath thickness, and macroscopic α’ texture intensity are examined. Second, the relationship between α’ lath thickness and Vickers microhardness (HV0.5) is evaluated using a Hall–Petch relationship. Finally, residual stress results under different processing conditions are compared, and the effects of scanning speed and hatch distance on the variations in peak and bulk residual stresses are discussed. This work offers deeper insight and practical guidance for parameter optimization in additive-manufactured titanium alloys, beyond conventional VED-based frameworks.

## 2. Materials and Methods

### 2.1. LPBF Fabrication with Different Process Parameters

**Figure 1:**
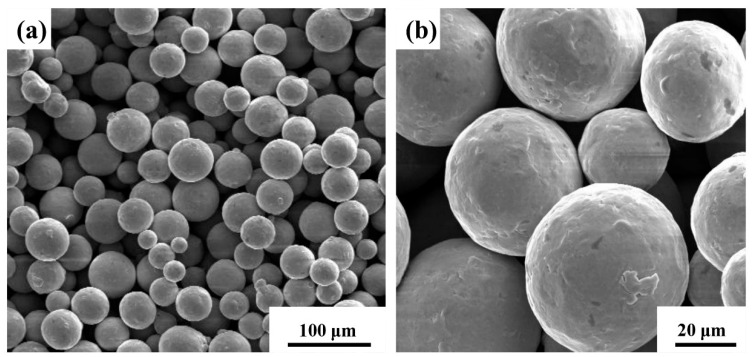
(**a**,**b**) The morphologies of Ti64 powder.

The Ti64 powder was provided by Jiangsu Jinwu New Material Co., Ltd. (Taizhou, China), with a particle size in the range of 20–53 μm and particle sizes of 22.8 μm (D10), 36 μm (D50), and 53.5 μm (D90) (Figure 1). The chemical compositions of Ti64 alloy powder are shown in Table 1.

**Figure 2:**
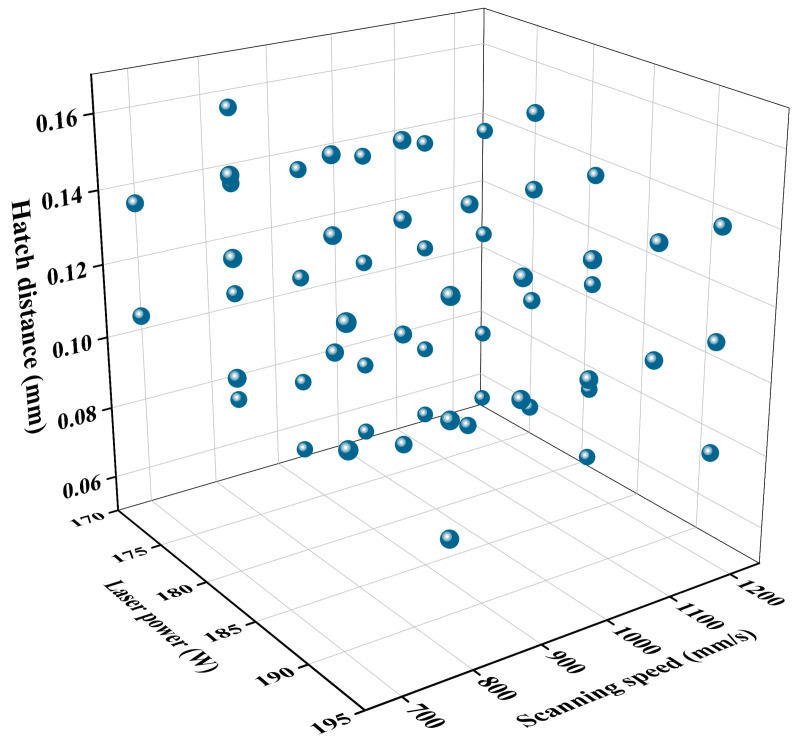
Distribution of process parameters for all 56 samples with designed process parameters.

All Ti64 specimens were produced by LPBF on an AmPro SP100^®^ system (Beifeng, Suzhou, China) with a 200 W Yb:YAG fiber laser (1067 nm) and a 60 µm beam diameter. All specimens were built along the Z direction (vertical to the build plate). A stripe scanning strategy was applied, with the scan vectors rotated by 67° between consecutive layers. All samples were built in a high-purity argon environment, with the oxygen concentration controlled below 1000 ppm to minimize oxidation during processing. A laser power (*P*) range of 173–193 W was selected for LPBF Ti64 alloy to minimize molten pool fluctuations and maintain stable processing conditions. The processing window was determined based on the machine capability and stable build conditions. This small range allowed the differences in microstructure and mechanical properties to be attributed mainly to process parameters rather than process instability. The scanning speed ranged (*v*) from 664 to 1200 mm/s, the hatch distance (*h*) varied from 0.06 to 0.16 mm, and the layer thickness (*t*) was 30 μm. Specifically, for each laser power setting (173, 183, and 193 W), the scanning speed was varied from 664 to 1200 mm/s at fixed hatch distances of 0.06, 0.08, 0.11, 0.14, and 0.16 mm. After completing one hatch distance series, the hatch distance was adjusted to the next value, and the same range of scanning speeds was repeated. These parameters covered different energy inputs, thermal history, and solidification dynamics, resulting in a total of 56 parameters detailed in Figure 2. The parameters are provided in Table S1 (Supplementary Materials). Optical microstructure characterization and microhardness tests were carried out in these 56 samples.

### 2.2. Microstructural Characterization

All the samples were mechanically ground with SiC papers (220# to 3000#), followed by polishing with oxide polishing suspension (OPS). For microstructural characterization, Kroll’s reagent (1.0 wt.% HF, 4.0 wt.% HNO_3_, and 95.0 wt.% H_2_O) was used to etch the polished surfaces for 20 s. The average relative density of all samples was 99.83%, measured by image analysis on polished XY-plane cross-sections (parallel to the build plate). For each set of samples, 20 optical micrographs at 50× magnification were acquired, and the density was calculated using ImageJ 1.53k.

The chemical composition of the powder was obtained using an Avio 500 ICP-OES (N0810010, PerkinElmer, Shelton, CT, USA). The optical microscopy (OM) images were acquired by the ZEISS Imager M2m optical microscope (ZEISS, Oberkochen, Germany). Scanning electron microscope (SEM) micrographs were obtained using Zeiss Gemini300 (ZEISS, Oberkochen, Germany) with a working voltage of 15 kV and a working distance of 20.7 mm. Backscattered secondary electron (BSE) micrographs were obtained using Zeiss Gemini300 with a working voltage of 15 kV and a working distance of 5.1–6.4 mm. ZEISS Gemini500 with an Oxford Symmetry EBSD detector (Oxford Instruments, Abingdon, UK) was used for electron backscatter diffraction at a working voltage of 20 kV, current of 15 nA, and a step size of 0.35 μm. EBSD was used primarily for crystallographic orientation and texture analyses, whereas the α/α’ lath morphology and lath width were measured and quantified from the BSE micrographs. AztecCrystal 2.1 was used to analyze the EBSD datasets. The MATLAB 2023a-based crystallographic toolkit MTEX (v5.11.2), together with the ORTools extension (v2.3.0), was used to conduct the intervariant pair analysis [51]. The twelve α’ variants were determined by ORTools from the reconstructed parent β orientations and are denoted as Variants 1–12. The macrotextures were obtained by X-ray diffraction, using Rigaku Smartlab (Rigaku, Tokyo, Japan) at a working voltage of 45 kV and a current of 200 mA. Texture data were collected over a 2θ range of 32–82° with a step size of 0.05°. The acquired diffraction data were post-processed in Python 3.14 before being visualized in Origin. The prior-β grain sizes were measured from OM images by using ImageJ, which were calculated from 15 measured images for each sample. The α’ martensite lath thickness was measured from BSE images using the linear intercept method specified in the GB/T 6394-2017 [52]. For each sample, at least *n* ≥ 300 intercepts were collected to ensure statistical reliability.

### 2.3. Microhardness and Residual Stress Tests

Samples were polished before microhardness testing. A Duramin-A300 tester (Struers, Ballerup, Denmark) was used to measure Vickers microhardness. A load of 500 g (HV0.5) was applied with a dwell time of 10 s. Five indentations were performed for each sample to ensure consistency.

Residual stress measurements of the as-built samples were conducted using the hole-drilling method. As shown in Figure S1 (Supplementary Materials), 23 × 23 × 10 mm^3^ samples were prepared. The measurements were carried out using a Restan MTS3000 system (SINT Technology, Calenzano, Italy) in accordance with the ASTM E837-13 standard [53]. A high-speed pneumatic drill with a 1.8 mm diameter bit was employed, together with a type-B three-element strain gauge rosette (0°/45°/90°) bonded at the sample center. Incremental drilling was conducted to a maximum depth of 1.2 mm, with a total of 48 steps and an average increment of 0.025 mm per step. The calculations were performed based on the elastic constants of Ti64 alloy, assuming an elastic modulus (E) of 110 GPa and a Poisson’s ratio (ν) of 0.32. The strain rosette was bonded on the top surface (the X-Y plane parallel to the build plate). The coordinate system was defined such that Z is the build direction and X is the scanning direction. The rosette 0° direction was aligned with the scan vector (X), and 90° corresponds to the in-plane transverse direction (Y). Due to the limited sample height (10 mm), which is insufficient for stable rosette attachment on the side surface, the stress along the build direction was not characterized.

## 3. Results

### 3.1. Prior-β Grain Morphologies

**Figure 3:**
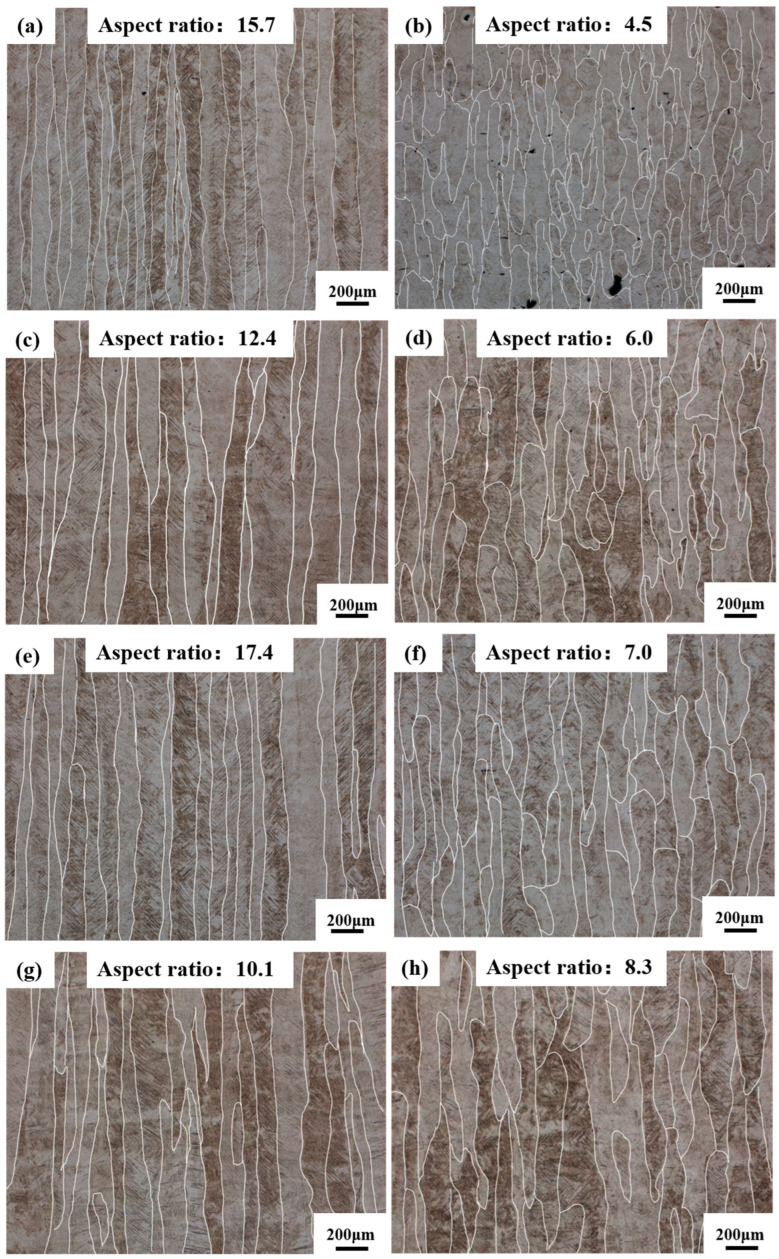
The evolution of prior-β grain morphology and aspect ratios under different process parameters. (**a**) *P* = 173 W, *v* = 1200 mm/s, *h* = 0.08 mm; (**b**) *P* = 173 W, *v* = 1200 mm/s, *h* = 0.14 mm; (**c**) *P* = 183 W, *v* = 1100 mm/s, *h* = 0.08 mm; (**d**) *P* = 183 W, *v* = 1100 mm/s, *h* = 0.11 mm; (**e**) *P* = 193 W, *v* = 800 mm/s, *h* = 0.14 mm; (**f**) *P* = 173 W, *v* = 800 mm/s, *h* = 0.14 mm; (**g**) *P* = 193 W *v* = 664 mm/s, *h* = 0.14 mm; (**h**) *P* = 183 W, *v* = 664 mm/s, *h* = 0.14 mm.

As illustrated in Figure 3, with varying processing parameters, the prior-β grain structure exhibited the transition from columnar grains to equiaxed grains. The morphologies of prior-β grains were dominated by columnar grains with well-defined boundaries, growing epitaxially along the build direction, with lengths exceeding 1.00 mm. The average columnar grain widths of these samples ranged from 124.59 to 143.52 μm. Elongated columnar grains had a high average aspect ratio of 10.1–17.4 measured by OM images with lower magnification in Figure 3a,c,e,g. In contrast, after altering the parameters by increasing the hatch distance and scanning speed, the prior-β grains exhibited more fragmented morphologies, which consisted of equiaxed grains alongside ‘less elongated’ columnar grains (Figure 3b,d,f,h). Concurrently, the average width ranged from 130.95 to 134.42 μm. The prior-β grains showed a lower average aspect ratio of 4.5–8.3. With the process parameters of *P* = 193 W, *v* = 800 mm/s, and *h* = 0.14 mm, columnar grains with the largest aspect ratio (17.4) and average width of 142.10 μm had been formed. Conversely, with the process parameters of *P* = 173 W, *v* = 1200 mm/s, and *h* = 0.14 mm, prior-β grains exhibited the smallest aspect ratio (4.5), with an average grain width of 124.26 μm.

**Figure 4:**
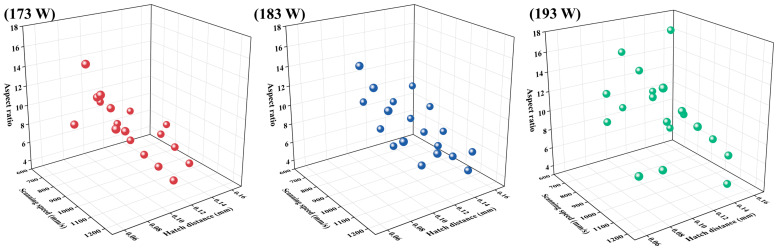
Aspect ratio of prior-β grains with different scanning speeds and hatch distances under laser powers of 173 W, 183 W, and 193 W. Each subplot corresponds to one parameter combination and shows the distribution of aspect ratios across all 56 processing conditions.

The aspect ratios of prior-β grains with the 56 different process parameters are presented in Figure 4. At *P* = 173 W, the microstructure exhibited a broad spread in grain morphology, with aspect ratios spanning from 4.5 to 16.5. The most equiaxed grains occurred at a scanning speed of 1200 mm/s combined with a hatch distance of 0.14 mm. In contrast, markedly elongated grains (aspect ratio > 15) were concentrated in the regime of high scanning speeds (>950 mm/s) together with a small hatch distance (<0.08 mm). When the scanning speed exceeded 1000 mm/s, and the hatch distance became large (>0.12 mm), the grains reverted to a less elongated shape, with aspect ratios falling below 6. Increasing the laser power to 183 W shifted the parameter space associated with highly elongated grains. Aspect ratios between 5.3 and 15.2 were recorded, with the highest values emerging mainly at moderate scanning speeds (700–1050 mm/s) coupled with small hatch distances (<0.08 mm). In regions of both high scanning speed (>1100 mm/s) and large hatch distance (>0.12 mm), the grain morphology approached the equiaxed state, yielding aspect ratios below 6. At *P* =193 W, most parameters produced prior-β grains with higher aspect ratios compared with the 173 W and 183 W conditions. Aspect ratios ranged from 7.5 to 17.4, with most samples exhibiting values above 8. A broad processing window, which includes 750–830 mm/s scanning speeds and hatch distances of 0.08–0.14 mm, produced average aspect ratios exceeding 10. The most pronounced elongation (aspect ratio > 15) occurred at relatively slow scanning speeds (<750 mm/s) combined with moderate hatch distances (<0.11 mm). Conversely, less elongated grains (aspect ratio < 8) were observed when the scanning speed rose above 900 mm/s, together with either a small (<0.09 mm) or large (>0.13 mm) hatch distance.

### 3.2. α’ Phase Characterization

**Figure 5:**
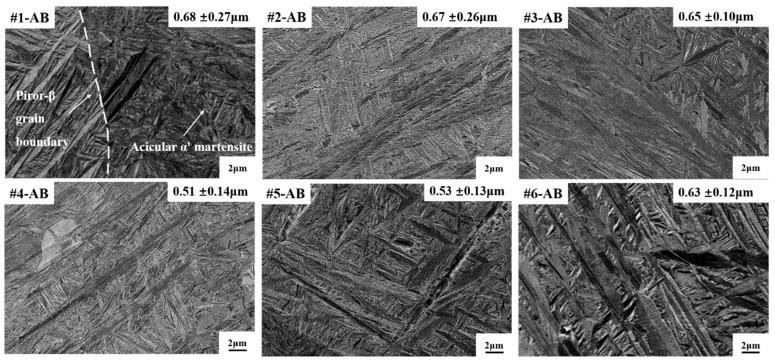
BSE micrographs of the six selected samples in as-built (AB) states showing the α’ martensite microstructures. The average α’ lath thickness was labeled. (#1) *P* = 173 W, *v* = 800 mm/s, *h* = 0.08 mm; (#2) *P* = 193 W, *v* = 800 mm/s, *h* = 0.08 mm; (#3) *P* = 193 W, *v* = 1200 mm/s, *h* = 0.08 mm; (#4) *P* = 173 W, *v* = 1200 mm/s, *h* = 0.14 mm; (#5) *P* = 183 W, *v* = 664 mm/s, *h* = 0.11 mm; (#6) *P* = 183 W, *v* = 800 mm/s, *h* = 0.11 mm.

BSE images revealed the evolution of α’ martensites in as-built (AB) states from #1 to #6 (Figure 5). All samples displayed dense networks of acicular α’ martensite, with lath widths between 0.51 and 0.68 μm. These α’ martensite laths form a basket-weave structure within the prior-β grains and exhibited a relatively uniform distribution. Among the AB samples, #1 contained the coarsest α’ martensite laths (0.68 ± 0.27 μm), accompanied by clearly defined prior-β boundaries across which martensite orientation changes noticeably. In contrast, #4 presented the finest laths (0.51 ± 0.14 μm), characterized by a relatively more uniform distribution.

**Figure 6:**
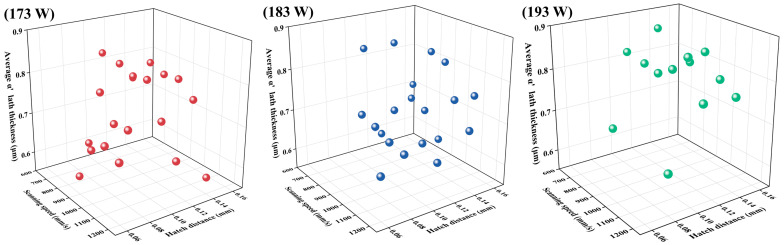
α’ martensite lath thickness with different scanning speeds and hatch distances under laser powers of 173 W, 183 W, and 193 W. Each subplot corresponds to one parameter combination and shows the distribution of α’ martensite lath thickness across all 56 processing conditions.

As shown in Figure 6, the variations in α’ martensite lath thickness across all processing conditions revealed distinct coarsening and refinement trends at each laser power level. At *P* = 173 W, the measured lath thicknesses span approximately 0.51–0.88 μm. Finer α’ laths (≤0.65 μm) generally occurred at relatively high scanning speeds (>800 mm/s) combined with hatch distances of either 0.06–0.08 mm or 0.12–0.14 mm. The thinnest α’ laths (0.51 μm) appeared at the combination of 1200 mm/s scanning speed and a hatch distance of 0.14 mm. At *P* = 183 W, α’ martensite laths fall within a narrower range (0.63–0.83 μm). Coarser morphologies (>0.75 μm) were mainly associated with lower scanning speeds (664–900 mm/s) together with hatch distances of 0.11–0.14 mm or with high hatch distances (>0.14 mm) at moderate scanning speeds (800–1200 mm/s). Slightly refined α’ martensite laths (<0.65 μm) may still emerge under two sets of conditions: high scanning speeds (1000–1100 mm/s) paired with large hatch distances (0.11–0.14 mm) and relatively slow scanning speeds (750–850 mm/s) combined with hatch distances of 0.10–0.12 mm. At *P* = 193 W, α’ martensite lath thickness became more uniform across the process window, with most values exceeding 0.65 μm. The finest α’ martensite laths (0.53 μm) were produced at 800 mm/s and a hatch distance of 0.14 mm. Slight refinement (~0.70 μm) was found when the hatch distance is reduced below 0.10 mm, and the scanning speed exceeds 800 mm/s. In contrast, larger hatch distances (>0.11 mm) combined with moderate-to-high scanning speeds (664–1200 mm/s) promoted noticeable coarsening, yielding α’ martensite lath thicknesses around 0.80 μm.

### 3.3. Texture and EBSD Characterization

Six process parameters (Samples #1–#6, Table 2) were chosen for further detailed characterization, including EBSD and XRD characterization, and residual stress measurements. These parameters spanned the investigated ranges of scanning speed, hatch distance, and laser power, covering typical conditions from low to high scanning speeds and from small to large track overlap.

**Figure 7:**
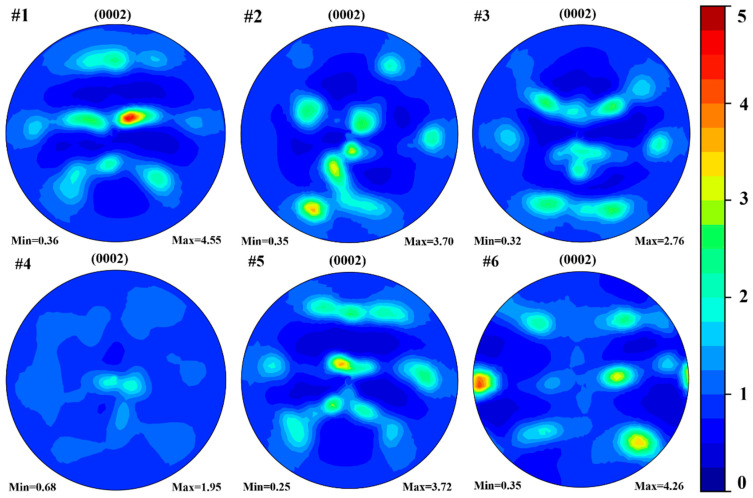
(0002) pole figures obtained from XRD measurements for the same samples. The build direction (BD) corresponds to the center of each pole figure.

The (0002) pole figures of the six selected samples with different process parameters measured from XRD exhibited pronounced differences in both the texture types and sharpness, as illustrated in Figure 7, Sample #1, with the process parameters of *P* = 173 W, *v* = 800 mm/s, and *h* = 0.08 mm exhibiting a relatively sharp texture (with the max texture intensity = 4.55) and indicating the alignment of the (0002) plane 45° to the build direction, which was the solidification texture formed during the LPBF process. Similar solidification texture with different sharpness could be found in Samples #3, #5, and #6, with maximum texture intensities of 2.76, 3.72, and 4.26, respectively. Sample #2, with the process parameters of *P* = 193 W, *v* = 800 mm/s, and *h* = 0.08 mm, exhibited a texture with the max texture intensity of 3.70 and (0002) crystallographic planes perpendicular to the LPBF fabrication direction, indicating the texture deviated from the solidification texture. Sample #4, with the process parameters of *P* = 173 W, *v* = 1200 mm/s, and *h* = 0.14 mm, exhibited the macrotexture close to random (with the max texture intensity of 1.95), characterized by a relatively weak concentration of (0002) poles perpendicular to the fabrication direction, which deviated from the solidification texture and could be considered close to random.

**Figure 8:**
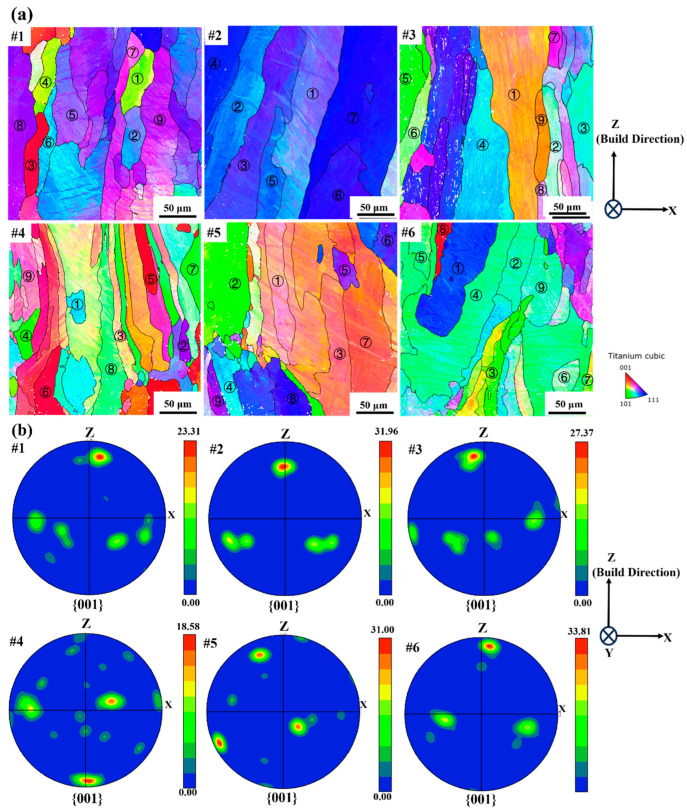
(**a**) Inverse pole figure orientation maps of reconstructed β grains and orientation maps of the 6 selected samples. (**b**) Corresponding {001} β orientation distribution maps (pole figures).

As shown in Figure 8a, the inverse pole figure (IPF) maps of the six samples revealed columnar prior-β grains aligned with the build direction, but with distinct differences in morphology and grain orientation, which was consistent with the OM characterization. Sample #1 exhibited more irregular grains, characterized by locally interrupted boundaries, in which the reconstructed prior-β grains failed to form continuous contours and instead appeared as randomly segmented boundary fragments, with a KAM of 0.71 ± 0.44°. In contrast, samples #2, #3, #5, and #6 displayed long and well-aligned columnar β grains with relatively consistent orientations in the prior-β grains, with KAMs of 0.52 ± 0.35°, 0.52 ± 0.37°, 0.57 ± 0.38°, and 0.69 ± 0.45°, respectively. Sample #4 showed the most fragmented morphology, consisting of a mixture of equiaxed and less elongated grains with highly discontinuous boundaries. These reconstructed β boundaries were fragmented due to local orientation deviations and therefore failed to develop into continuous contours, with a KAM of 0.51 ± 0.34°.

The {001} β pole figures from the reconstructed prior-β EBSD datasets showed that Samples #1, #3, and #6 exhibited the most poles aligned close to the build direction, which was the typical <001>β solidification texture (Figure 8b). Samples #2 and #5 also showed {001} alignment toward the build direction, though with lower intensity and broader distribution, deviating from the solidification texture. Despite the dominance of solidification texture in Sample #4, a relatively weak texture with more scattered poles, as revealed by the {001} alignments, could be identified.

**Figure 9:**
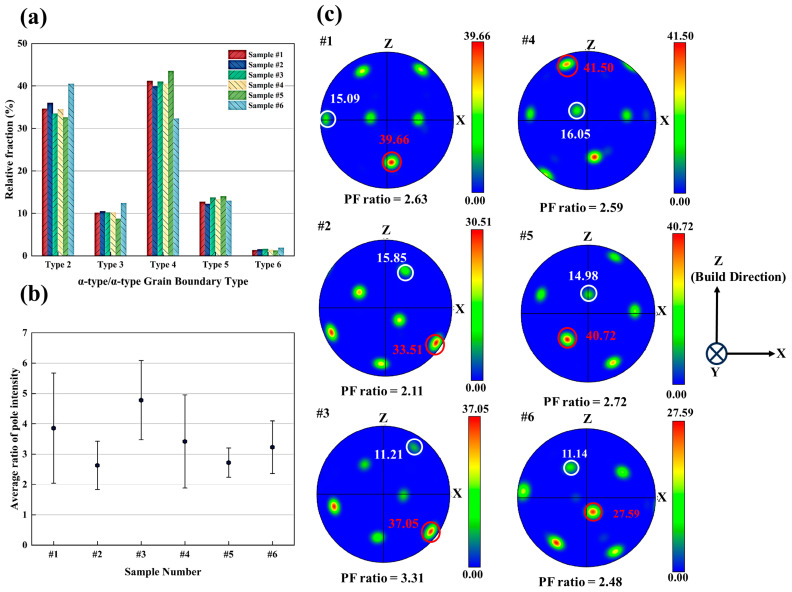
(**a**) Relative fractions of α-type/α-type boundary misorientation types (Types 2–6) for Samples #1–#6; (**b**) average {0001} pole–figure intensity ratio (max/min) calculated from representative prior-β grains (Grains ①–⑨) in each sample; (**c**) {0001} pole figures of Grain ① from the 6 samples.

Table 3 summarizes the five typical α-type/α-type boundary types, and Figure 9a shows the relative fractions of the α/α boundary misorientation types (Type 2–6) for Samples #1–#6. Samples #1–#5 exhibited similar distributions, with Type 4 being the most frequent boundary type (≈40–44%), followed by Type 2 (≈32–36%). Types 3 and 5 appeared with lower fractions, ranging from about 12% to 14%, while Type 6 remained below 2% in all five samples. In Sample #6, Type 2 became the most frequent boundary type (40.5%), whereas the fraction of Type 4 decreased to 32.3%.

The average {0001} pole–figure intensity ratios (max pole intensity/min pole intensity) calculated from seven to nine representative prior-β grains (Grains ①–⑨) in each sample are summarized in Figure 9b. The pole–figure intensity ratio provided a quantitative measure of the orientation clustering among α-type variants within a single grain. A higher intensity ratio indicates a stronger variant selection, while a lower ratio reflects the activation of multiple variants [54]. Sample #3 exhibited the highest average ratios, 4.78. The values for Samples #2 and #5 showed the lowest intensity ratios, 2.63, 2.67, and 2.72. Samples #1, #4, and #6 had intensity ratios of 3.86, 3.42, and 3.23. The {0001} pole figures of Grain ① from the selected six samples are shown in Figure 9c for a direct comparison of the α-type variant selection within representative prior-β grains. In all six samples, the {0001} pole figures displayed pronounced nonuniformity in pole intensity. The maximum pole intensity values ranged from 27.59 to 41.50, and the minimum intensity values were considerably lower (11.14–16.05) in every sample. The pole intensity ratio varied among the six samples. For Grain ① in Sample #3, the ratio reached the highest value of 3.31, while Samples #2 and #6 exhibited the lowest ratios of 2.11 and 2.48. The remaining samples showed intermediate ratios of 2.5–3 (2.63 in Sample #1, 2.59 in Sample #4, and 2.72 in Sample #5).

**Table 3:** Five typical α-type/α-type boundary types of LPBF Ti64 alloy [55].

Type	Orientation Relationships
2	$60{}^{\circ}/<11\bar{2}0$>
3	$60.83{}^{\circ}/<\bar{10}\;\bar{7}\;1\;7\;3$>
4	$63.26{}^{\circ}/<\bar{10}\;55\bar{3}$>
5	$90{}^{\circ}/<\bar{7}\;17\bar{\;10}\;0$>
6	10.53°/<0001>

**Figure 10:**
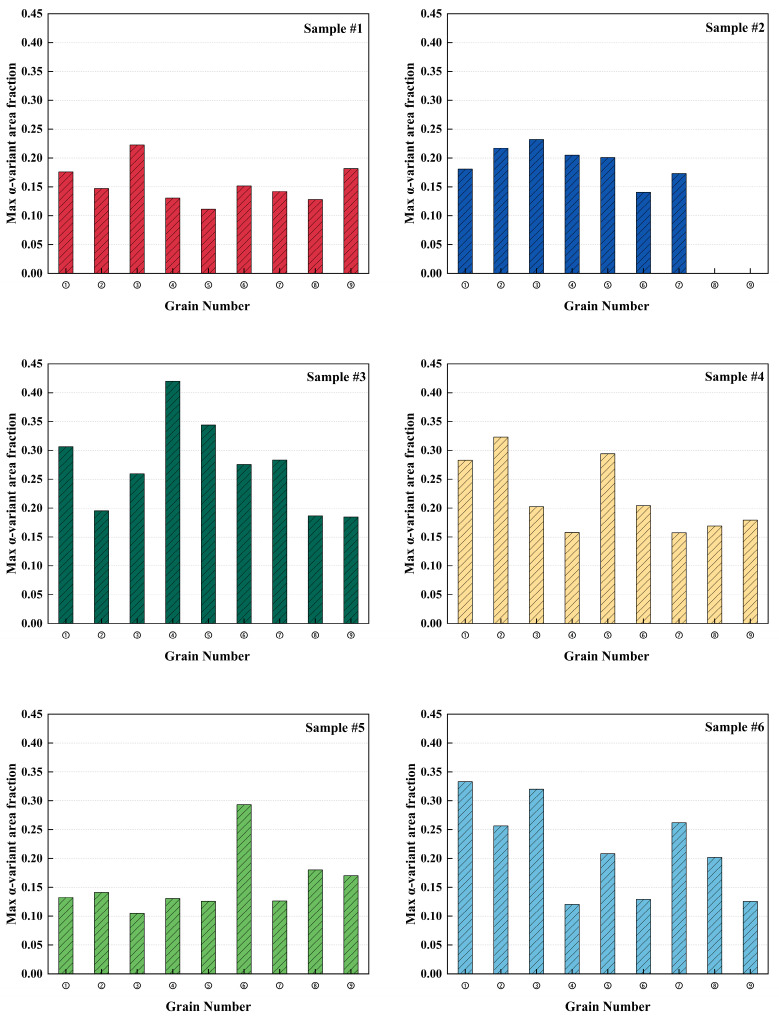
Distribution of max α-variant area fraction for Samples #1–#6. Each column corresponds to one reconstructed prior-β grain (Grains ①–⑨) in each sample.

To quantitatively characterize the extent of α-variant selection at the prior-β grain scale, the maximum area fractions among the 12 α-type variants within each reconstructed prior-β grain were processed statistically. As shown in Figure 10, the distributions of the dominant-variant area fraction showed distinctive differences among the samples, indicating that the process parameters influenced the grain-scale variant-selection. For Sample #1, the values were mainly distributed in the range of ~0.11–0.18, with occasional values approaching ~0.22. Sample #2 showed a more concentrated distribution of ~0.14–0.23. Sample #3 exhibited the widest range (~0.18–0.42), with the maximum reaching ~0.42, whereas Sample #4 was mainly distributed in ~0.15–0.33. For Sample #5, the values ranged from 0.10 to 0.29, with most data points between ~0.10 and 0.18. Sample #6 ranged from 0.12 to 0.34, with multiple data points exceeding 0.20.

**Figure 11:**
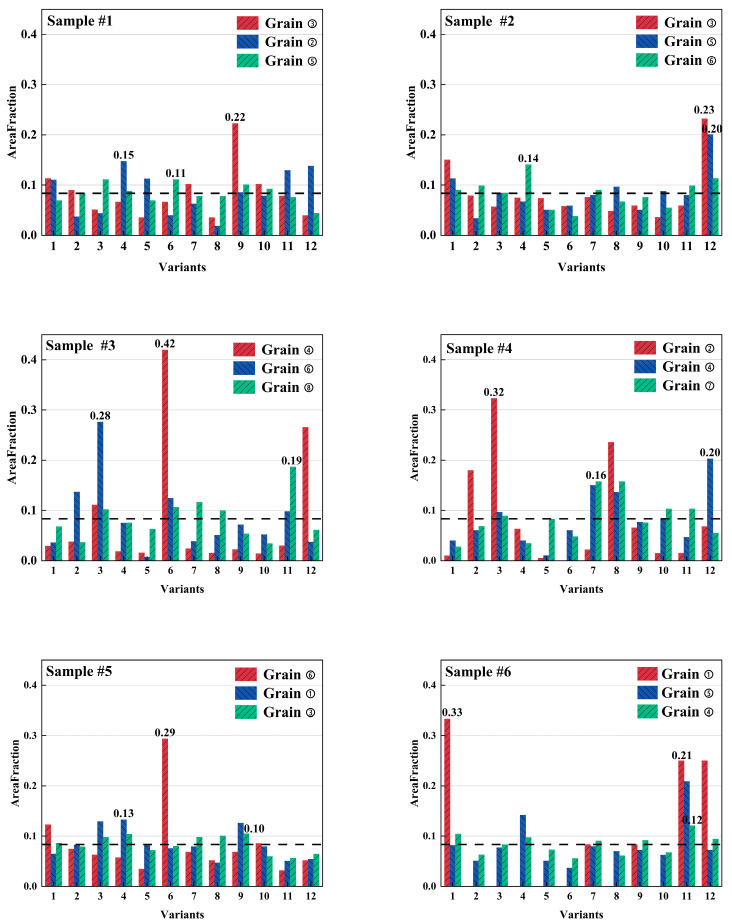
Area-fraction distributions of the 12 α-type variants in representative reconstructed prior-β grains for Samples #1–#6. For each sample, three grains were selected: the grain type with the maximum dominant α-type variant area fraction, the grain type with the dominant α-type variant area fraction closest to the sample median, and the grain type with the minimum dominant α-type variant area fraction. The dashed line indicates the uniform baseline (1/12).

For each sample, variant fractions were calculated for all reconstructed prior-β grains (Grains ①–⑨), and the corresponding 12-variant area-fraction datasets are provided in Figures S2–S7 (Supplementary Materials). Figure 11 presented the area-fraction distributions of the 12 α-type variants for three selected reconstructed prior-β grains in Samples #1–#6 (grains with the maximum, median, and minimum dominant α-type variant area fractions). In all samples, the variant area-fraction distributions deviated markedly from the uniform baseline, indicating that nonuniform variant activation was commonly present at the single prior-β grain scale. Within each sample, the three grains exhibited a consistent difference: the grain with the largest dominant α-type variant area fraction typically showed a more pronounced dominant peak, and the median grain displayed an intermediate peak magnitude, whereas the grain with the smallest dominant α-type variant area fraction remained closer to the baseline with comparatively weaker fluctuations. Among all the samples, clear differences were observed: Sample #3 exhibited the most prominent peaks with multiple variants reaching elevated area fractions; Sample #6 showed similarly pronounced peaks; Sample #4 displayed intermediate peak magnitudes with several peaks above the baseline. In contrast, Samples #1, #2, and #5 were overall closer to the baseline, with more moderate peaks and distributions.

### 3.4. Microhardness and Residual Stress

**Figure 12:**
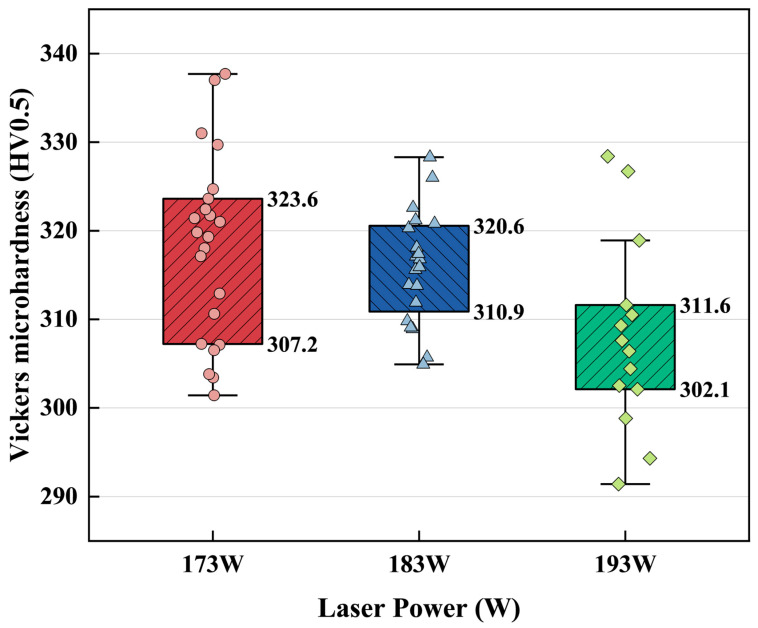
Box plots showing the distribution of Vickers microhardness (HV0.5) under different laser power conditions (173 W, 183 W, and 193 W).

The Vickers microhardness values of all samples fell within a relatively narrow interval of 291.4–337.7 ± 10.3 HV0.5, with only limited variation among the different processing conditions. As shown in Figure 12, under low laser power (173 W) parameters, the microhardness measurements ranged from 307.2 to 323.6 HV0.5. With moderate laser power (183 W) parameters, the data points became more clustered (310.9–320.6 HV0.5). Under high laser power (193 W) parameters, the microhardness values were distributed within a narrower band compared with the other conditions, ranging from 302.1 to 311.6 HV0.5. Overall, the microhardness remained largely consistent across the full parameters, indicating only minor sensitivity to the combinations of scanning speed and hatch distance.

**Figure 13:**
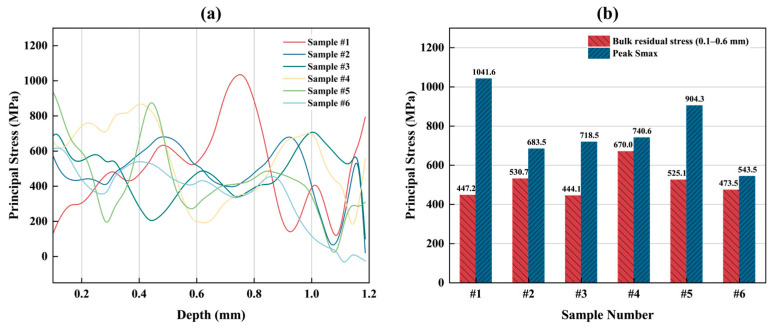
(**a**) Depth-resolved principal residual stress (Smax) profiles of the six selected samples; (**b**) comparison of bulk residual stress (0.1–0.6 mm average) and peak principal stress extracted from the profiles.

Residual stresses in LPBF are primarily thermally induced, resulting from steep thermal gradients and constrained thermal contraction during repeated layer-by-layer thermal cycling [49,50,51]. Figure 13a shows the depth-resolved principal residual stress profiles of the six selected samples obtained by the incremental hole-drilling method. All samples exhibited oscillatory, multi-peak distributions rather than a monotonic trend. Because all samples were manually ground and polished before testing, the near-surface residual stress (depth < 0.1 mm) may be affected by polishing. Therefore, only the residual stress values beyond 0.1 mm were considered reliable and used for comparison among samples. The bulk residual stress was defined as the average Smax within the 0.1–0.6 mm depth range, whereas the peak Smax was defined as the global maximum principal stress within the same valid depth range. As shown in Figure 13b, Sample #1 and Sample #5 exhibited high peak tensile stresses (1041.6 MPa and 904.3 MPa, respectively), whereas Sample #3 and Sample #6 showed low peaks. The bulk residual stress followed a different trend: Sample #4 exhibited the highest mean subsurface stress (670.0 MPa), while Sample #3 and Sample #6 again showed low values.

## 4. Discussion

### 4.1. Evolution of Microstructure Under Different Process Parameters

Variations in parameters substantially change the heat input, cooling rate, and melt-track overlap, and these factors directly determine the molten pool geometry and its stability [56,57,58,59,60,61,62,63]. The resulting changes in molten pool aspect ratio and track remelting continuity govern the solidification mode and ultimately the final microstructure [64].

In this study, different LPBF parameters produced pronounced variations in prior-β grain morphology, ranging from highly fragmented and nearly equiaxed grains to large and continuous columnar grains. Process parameters, including scanning speed, hatch distance, and laser power, determine the effective energy input and thereby affect the depth, width, and thermal stability of the molten pool [65]. A stable and directionally coherent thermal gradient allows layer-to-layer continuation of β growth, producing large and continuous columnar grains [66]. A deeper and more thermally stable molten pool maintains such heat flow and thus further supports continuous β epitaxy [16,54]. Therefore, the dependence of prior-β grain morphology on the process parameters fundamentally arises from their regulation of molten pool stability, which in turn governs continuous β epitaxial growth. This also accounts for the pronounced morphological variations in the β grains in this study: parameters with lower scanning speed (below 800 mm/s) and small hatch distances (0.06–0.08 mm) that can form stable molten pools tend to preserve the epitaxial front and produce coarser, more elongated β grains. In contrast, parameters associated with reduced overlap and accelerated cooling, such as higher scanning speeds (over 1200 mm/s) or larger hatch distances (over 0.10 mm), disrupt the stability of the growth front and result in more fragmented β grains [67].

Previous studies have shown that, with laser power and hatch distance constant, lower scanning speeds generally promote the formation of longer and more columnar prior-β grains, whereas higher scanning speeds tend to produce shorter and less elongated grains [68]. Although this trend is evident in Samples #2 and #3, which differ only in scanning speed, it does not extend to the full parameters because laser power, scanning speed, and hatch distance vary simultaneously. In fact, not all samples with only one parameter different exhibited similar grain morphologies. For example, samples fabricated at the same scanning speed and hatch distance but with different laser power may exhibit distinct prior-β grains (Figure 3). In addition, laser power plays an important role in molten pool behavior by influencing the local thermal conditions and molten pool dynamics in combination with other processing parameters [65]. It is obvious that molten pool stability cannot be described by VED alone, since varied parameters may produce distinct thermal conditions. This explains why samples processed with similar VEDs, such as Samples #3 and #6, exhibited markedly different prior-β morphologies. For Sample #3, the combination of higher laser power and smaller hatch distance promoted a more stable and continuous thermal environment, which supported sustained β epitaxial growth. In contrast, Sample #6, with lower laser power, scanning speed, and larger hatch distance, developed a less stable molten pool that disrupted β growth.

The α’ martensite laths also exhibited marked differences in thickness across the samples. Although α’ martensite originates from the transformation of the prior-β grains, its morphology is primarily controlled by the cooling rate and thermal accumulation rather than by epitaxial stability [69]. Equiaxed and columnar β grains with both coarse and fine α’ laths were identified, showing that α’ lath thickness is not directly determined by β grain morphology. High cooling rates typically result in fine α’ laths due to the limited growth time during solidification, whereas slower cooling associated with thermal accumulation facilitates the formation of thicker α’ laths, as commonly observed at lower scanning speeds and higher laser power [10]. Previous studies have shown that the lath thickness of α’ martensite in LPBF Ti64 alloy is primarily governed by the cooling rate, with scanning speed having the strongest influence on cooling behavior, while hatch distance plays a secondary role by affecting melt-track overlap and local thermal accumulation [69]. This is because the solidification cooling rate tends to increase with scanning speed and may follow an approximate power–law relationship, whereby changes in scanning speed can produce relatively large variations in cooling rate. The results of this study further support this view. Within the investigated processing window, α’ lath thickness was primarily controlled by scanning speed and showed a consistent refinement trend as scanning speed increased, regardless of laser power and hatch distance. Hatch distance had a secondary effect on α’ lath thickness, with larger hatch distances generally leading to thicker α’ laths at similar scanning speeds; however, this effect became weaker at higher scanning speeds. Within the investigated laser power range, laser power mainly regulated the overall level of α’ lath thickness across different parameters, rather than dominant control. Moreover, Samples #3 and #6, which had similar VED values, exhibited almost the same α’ lath thicknesses. This further supports the conclusion that the overall cooling rate, largely determined by the energy input, plays the dominant role in controlling α’ morphology instead of β grains.

### 4.2. Texture Development with Different LPBF Parameters

α-type variant selection primarily occurs during the nucleation and early growth stages of the β → α or β → α’ transformation [70]. Among the five α-type/α-type boundary types, Type 6, which corresponds to low-angle boundaries, is strongly suppressed under the rapid cooling conditions typical of LPBF. In contrast, high-angle Type 2 boundaries are promoted by substantial thermal stresses and cyclic reheat during LPBF, and their fraction increases under relatively lower cooling rates. Type 4 boundaries are favored by steep thermal gradients and highly directional solidification, and their prevalence increases with increasing cooling rate [71]. These trends are consistent with prior reports showing that high cooling rates and strongly directional thermal gradients, particularly in columnar prior-β grains, promote Type-4-dominated α-type/α-type boundary distributions [54,55,72]. In contrast, lower cooling rates or equiaxed prior-β grains tend to yield Type-2-dominated boundary populations and, in many cases, a stronger degree of variant selection [55]. In this study, Samples #1–#3 and #5 exhibited Type-4-dominated α/α boundaries, which can be attributed to the relatively high thermal gradients that promoted strong and directionally continuous prior-β epitaxy. Under such conditions, the β→α’ transformation is primarily governed by thermal-gradient-driven growth, favoring the formation of Type 4 boundaries. Although Sample #4 exhibited highly fragmented prior-β boundaries, its high cooling rate kept Type 4 as the dominant boundary type. In contrast, Sample #6 showed an increased fraction of Type 2 boundaries, which is associated with a reduced cooling rate and weakened epitaxial growth.

**Figure 14:**
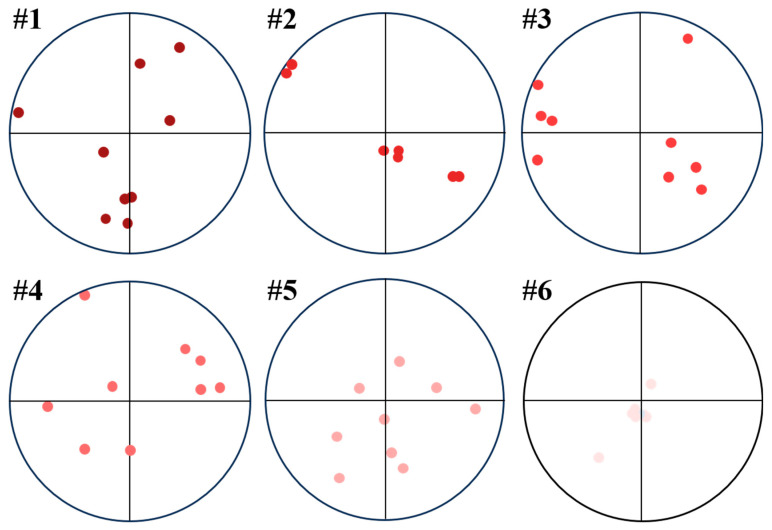
The maximum {0001} pole distributions for representative prior-β grains in the six samples identified in Figure 8. The {0001} pole with the highest intensity in the corresponding single-grain pole figure was re-plotted to illustrate the orientation distribution within each sample.

To further characterize and compare the variant selection in different samples, the positions of the {0001} poles with the highest intensities in the identified prior-β grains in Figure 8 were re-plotted as shown in Figure 14. Despite the strong variant selection of all six samples as indicated by the relatively high {0001} pole–figure intensity ratios, the crystallographic orientations of the preferred variants in different prior-β grains could be varied and could potentially lead to the weak texture, as shown by Sample #4. In contrast, the preferred variants of different prior-β grains in Samples #2 and #6 had very similar crystallographic orientations, which indicated sharp prior-β texture and strong variant selection with consistent variants formed. The weak texture in Sample #4, as shown by Figure 7, and the strong variant selection as indicated in Figure 9, indicated that the prior-β texture could be another factor determining the transformed α texture at room temperature.

In LPBF Ti64 alloy, columnar β grains commonly grow epitaxially along the <001>β direction, forming a characteristic solidification texture with the <001> orientations along the fabrication direction [66]. However, pronounced differences in the prior-β texture were observed between samples. In particular, the weak solidification texture with {001} poles deviated from the fabrication direction in Sample #4, indicating a significant reduction in the continuity of prior-β epitaxial growth. This suggests that, despite the thermal gradient along the fabrication direction, it is insufficient to sustain continuous β epitaxy. This leads to β grains in Sample #4 exhibiting the <001>β solidification texture with some orientation deviations. When the parameter promotes a stable molten pool geometry and a consistent thermal gradient, β grains are more likely to maintain continuous epitaxial growth along the fabrication direction, resulting in a sharp <001>β texture. In this study, such behavior was typically observed under higher laser power, which could cause enhanced thermal accumulation with relatively lower cooling rates. In contrast, insufficient melt-track overlapping and excessively rapid cooling could interrupt the epitaxial growth, produce dispersed crystallographic orientation distributions, and weaken the β solidification texture, which were more pronounced under relatively lower laser power and higher cooling rates.

**Figure 15:**
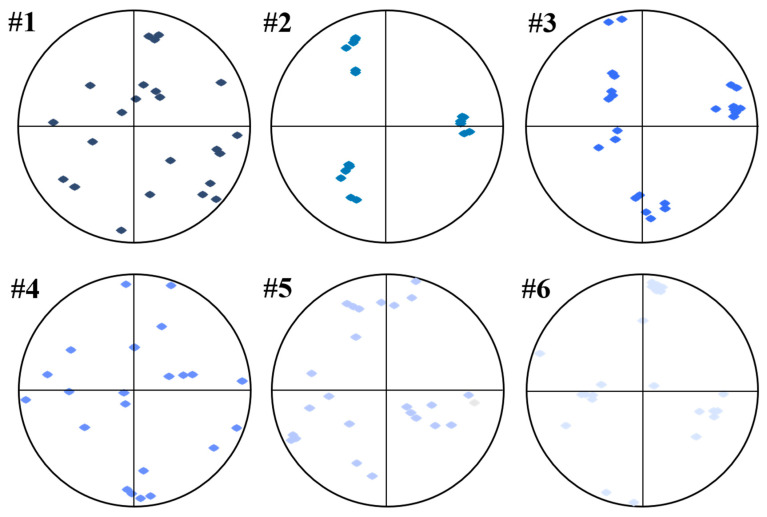
The maximum {001} pole distributions for representative prior-β grains in the six samples identified in Figure 8. The {001} pole with the highest intensity in the corresponding single-grain pole figure was re-plotted to illustrate the orientation distribution within each sample.

To further reveal the orientation coherence among prior-β grains within individual samples, the positions of the maximum {001} β poles of representative prior-β grains identified in Figure 8 were re-plotted, as shown in Figure 15. Significant differences in <001>β orientation distributions were observed among prior-β grains within the same sample. In Samples #2, #3, and #6, the maximum {001} β poles of different prior-β grains were concentrated, indicating a high orientation coherence, which is favorable for maintaining continuous β epitaxial growth along the fabricate direction. In contrast, Sample #4 exhibited a more dispersed distribution of {001} β pole positions among prior-β grains, reflecting pronounced orientation deviations.

The β solidification texture and α-type variant selection, therefore, play distinct yet coupled roles in determining the final α’ texture. A strong <001>β solidification texture enables effective orientation inheritance during the β → α’ transformation, which could lead to a sharp transformed α’ texture in LPBF titanium alloys with even moderate variant selection, like Samples #1 and #5. In the meantime, the variant selection in LPBF titanium alloys is considered extensive due to the rapid cyclic heating and high thermal stress [73,74]. In contrast, with a relatively weak β solidification texture, the orientations of the transformed α’ grains become scattered even with strong variant selection, which could lead to the α’ texture being close to random.

### 4.3. Relationship Between Mechanical Properties and Microstructure and Variations

**Figure 16:**
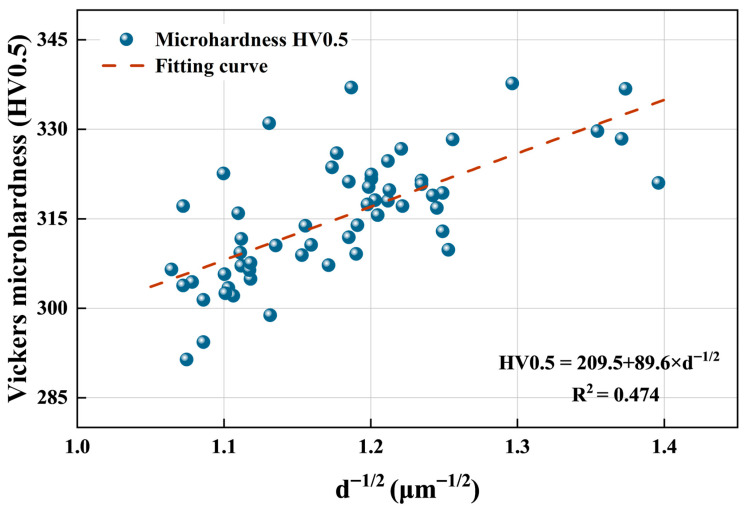
Hall–Petch relationship between the inverse square root of α’ martensite lath thickness (d^−1/2^) and Vickers microhardness (HV0.5).

The microhardness, as an indicator of resistance to plastic deformation, which often correlated with yield strength, could be correlated to the α’ martensite thickness with the Hall–Petch equation in this study, with a moderate correlation coefficient as shown in Figure 16 (R^2^ = 0.474). The grain refinement remains a dominant strengthening mechanism in LPBF Ti64 alloy within the present processing window. In the meantime, residual stress is another factor that could affect microhardness. Scanning speed and hatch distance play distinct roles, with peak residual stress being more sensitive to scanning speed due to heat accumulation and steep thermal gradients, whereas bulk residual stress is more strongly influenced by hatch distance through its effect on melt-track overlap and thermal distribution [75]. The residual stress obtained in this study is broadly consistent with these reported trends. Pronounced stress peaks were more frequently observed in samples processed at lower scanning speeds, reflecting the sensitivity of peak principal stress to local thermal accumulation. In contrast, samples fabricated with larger hatch distances exhibited elevated bulk residual stress levels, even when distinct stress peaks were absent, indicating that bulk residual stress is governed by the thermal cumulation across multiple melt tracks. Notably, samples with comparable VED values nevertheless exhibit markedly different peak and bulk residual stresses.

## 5. Conclusions

In this study, we systematically investigated the effect of laser power, scanning speed, and hatch distance on microstructural evolution, texture development, microhardness, and residual stress in LPBF Ti64 alloy. Firstly, different parameters produced pronounced variations in prior-β grain morphology, ranging from continuous columnar grains to highly fragmented grains together with changes in α’ lath thickness and macroscopic α’-texture intensity. Within the selected processing window, α’ lath thickness was dominated by scanning speed and generally refined with increasing scanning speed. A strong β solidification texture was required to develop a sharp macroscopic α’ (0002) texture, whereas α-type variant selection mainly enhances the texture intensity rather than determining it. Secondly, the microhardness showed a clear correlation with α’ lath refinement in a Hall–Petch equation, indicating that the microhardness variation is closely associated with changes in the α’ lath thickness across the investigated conditions. Finally, residual stress varied with parameters, where scanning speed predominantly affected peak residual stress and hatch distance governed bulk residual stress; this contrast highlights distinct parameter sensitivities for peak and bulk residual stress responses within the selected processing window.

## Figures and Tables

**Table 1:** Chemical compositions of Ti64 powder (wt.%).

Al	V	O	Fe	C	N	H	Ti
6.07	4.2	0.138	0.16	0.02	0.013	0.002	Bal.

**Table 2:** Process parameters of the six parameters selected for further investigation.

Sample Number	Laser Power (W)	Scanning Speed (mm/s)	Hatch Distance (mm)	Layer Thickness (μm)	Volumetric Energy Density (J/mm^3^)
#1	173	800	0.08	30	90.10
#2	193	800	0.08	30	100.52
#3	193	1200	0.08	30	67.01
#4	173	1200	0.14	30	34.33
#5	183	664	0.11	30	83.57
#6	183	800	0.11	30	69.32

## Data Availability

The original contributions presented in this study are included in the article/Supplementary Materials. Further inquiries can be directed to the corresponding authors.

## Supplementary Materials

The following supporting information can be downloaded at: https://www.mdpi.com/article/10.3390/ma19061049/s1, Figure S1: Samples of residual stress test. Red dots represent test points of 6 samples; Figure S2–S7: The corresponding 12-variant area-fraction of the reconstructed prior-β grains (Grains ①–⑨) in sample #1–#6; Table S1: The process parameters of all the 56 samples.

## References

[1] Kumar P., Prakash O., Ramamurty U. (2018). Micro-and meso-structures and their influence on mechanical properties of selectively laser melted Ti-6Al-4V. Acta Mater..

[2] Simonelli M., Tse Y.Y., Tuck C. (2014). Effect of the build orientation on the mechanical properties and fracture modes of SLM Ti-6Al-4V. Mater. Sci. Eng. A.

[3] Zhang L., Zhu H., Zhang S., Wang G., Zeng X. (2019). Fabricating high dimensional accuracy LPBFed Ti6Al4V part by using bi-parameter method. Opt. Laser Technol..

[4] Brika S.E., Letenneur M., Dion C.A., Brailovski V. (2020). Influence of particle morphology and size distribution on the powder flowability and laser powder bed fusion manufacturability of Ti-6Al-4V alloy. Addit. Manuf..

[5] Feng Z., Wang G., Hao Z., Wang Y., Tan H., Fan W., Dang M., Zhang S., Chen Y., Peng Y. (2024). Influence of scale effect on surface morphology in laser powder bed fusion technology. Virtual Phys. Prototyp..

[6] DebRoy T., Wei H.L., Zuback J.S., Mukherjee T., Elmer J.W., Milewski J.O., Beese A.M., Wilson-Heid A., De A., Zhang W. (2018). Additive manufacturing of metallic components—Process, structure and properties. Prog. Mater. Sci..

[7] Jamhari F.I., Foudzi F.M., Buhairi M.A., Sulong A.B., Mohd Radzuan N.A., Muhamad N., Mohamed I.F., Jamadon N.H., Tan K.S. (2023). Influence of heat treatment parameters on microstructure and mechanical performance of titanium alloy in LPBF: A brief review. J. Mater. Res. Technol..

[8] Chen Q., Thouas G.A. (2015). Metallic implant biomaterials. Mater. Sci. Eng. R Reports.

[9] Rani S.U., Kesavan D., Kamaraj M. (2023). Evaluation of influence of microstructural features of LPBF Ti-6Al-4 V on mechanical properties for an optimal strength and ductility. J. Alloys Compd..

[10] Cao S., Zou Y., Lim C.V.S., Wu X. (2021). Review of laser powder bed fusion (LPBF) fabricated Ti-6Al-4V: Process, post-process treatment, microstructure, and property. Light Adv. Manuf..

[11] Kang C.W., Fang F.Z. (2018). State of the art of bioimplants manufacturing: Part I. Adv. Manuf..

[12] Tan X., Kok Y., Tan Y.J., Descoins M., Mangelinck D., Tor S.B., Leong K.F., Chua C.K. (2015). Graded microstructure and mechanical properties of additive manufactured Ti-6Al-4V via electron beam melting. Acta Mater..

[13] Cepeda-Jiménez C.M., Potenza F., Magalini E., Luchin V., Molinari A., Pérez-Prado M.T. (2020). Effect of energy density on the microstructure and texture evolution of Ti-6Al-4V manufactured by laser powder bed fusion. Mater. Charact..

[14] Yao Z., Yang T., Yang M., Jia X., Wang C., Yu J., Li Z., Han H., Liu W., Xie G. (2022). Martensite colony engineering: A novel solution to realize the high ductility in full martensitic 3D-printed Ti alloys. Mater. Des..

[15] Li Z., Zhou X. (2026). Recent Advancements in Microstructure Control and Performance Optimization of Titanium Alloys via Powder Bed Fusion. Adv. Eng. Mater..

[16] Ni C., Zhu J., Zhang B., An K., Wang Y., Liu D., Lu W., Zhu L., Liu C. (2025). Recent advance in laser powder bed fusion of Ti–6Al–4V alloys: Microstructure, mechanical properties and machinability. Virtual Phys. Prototyp..

[17] Qian C., Zhang K., Zhu J., Ying L., Liu Y., Liu J., Liu J., Yang Y., Wang H. (2025). Effect of processing parameters on the defects, microstructure, and property evaluation of Ti-6Al-4V titanium alloy processed by laser powder bed fusion. AIP Adv..

[18] Chong Y., Bhattacharjee T., Yi J., Shibata A., Tsuji N. (2017). Mechanical properties of fully martensite microstructure in Ti-6Al-4V alloy transformed from re fi ned beta grains obtained by rapid heat treatment (RHT). Scr. Mater..

[19] Nguyen H.D., Pramanik A., Basak A.K., Dong Y., Prakash C., Debnath S., Shankar S., Jawahir I.S., Dixit S., Buddhi D. (2022). A critical review on additive manufacturing of Ti-6Al-4V alloy: Microstructure and mechanical properties. J. Mater. Res. Technol..

[20] Liu Y., Liu J., Zhang H., Yang Y., Zhu Y. (2025). Martensite decomposition and its effect on mechanical performance in laser powder bed fusion Ti–6.5Al–2Zr–1Mo–1V alloy. Mater. Sci. Eng. A.

[21] Yang Z., Qiu Y., Zhu J., Huang Z., Dai S., Yang W., Liu M., Wang G., Yan X., Huang W. (2025). Comparative study of LPBF Ta–Ti alloy: Microstructural evolution and deformation behavior. J. Mater. Res. Technol..

[22] Somlo K., Poulios K., Funch C.V., Niordson C.F. (2021). Anisotropic tensile behaviour of additively manufactured Ti-6Al-4V simulated with crystal plasticity. Mech. Mater..

[23] Bao Y., Yang X., Meng F., Yang Y., Chu Q., Wang M., Jenssen A. (2024). Effects of irradiation and stress on crack initiation behavior of 316SS flux thimble tubes in PWR environment. Corros. Commun..

[24] Liu F., Shi J., Huang J., Xu G., Guo Q., Xu J., Meng F. (2025). Influence of secondary phases on the corrosion behavior of IN718 in high-temperature pressurized water. Corros. Commun..

[25] Shan H., Zhang T., Yuan Y., Guo Q., Liu Z., Liu F., Xu J. (2024). Effects of dissolved oxygen on the corrosion-related unidentified deposit formed of 304 SS in the flow accelerated zone under the simulated secondary water chemistry. Corros. Commun..

[26] Xie J., Ma X., Kuang W. (2024). Improving the corrosion resistance of FeCrAl alloy in high temperature hydrogenated water through gaseous pre-oxidation. Corros. Commun..

[27] Bordbar-khiabani A., Gasik M. (2023). Electrochemical behavior of additively manufactured patterned titanium alloys under simulated normal, inflammatory, and severe inflammatory conditions. J. Mater. Res. Technol..

[28] Kaschel F.R., Celikin M., Dowling D.P. (2020). Effects of laser power on geometry, microstructure and mechanical properties of printed Ti-6Al-4V parts. J. Mater. Process. Technol..

[29] Joy A., Frederico I.B., Saranarayanan R.K., Wajira R., Browne D.J., Dowling D.P. (2023). The influence of a large build area on the microstructure and mechanical properties of PBF–LB Ti–6Al–4 V alloy. Int. J. Adv. Manuf. Technol..

[30] Mishurova T., Artzt K., Rehmer B., Haubrich J., Ávila L., Schoenstein F., Serrano-Munoz I., Requena G., Bruno G. (2021). Separation of the impact of residual stress and microstructure on the fatigue performance of LPBF Ti-6Al-4V at elevated temperature. Int. J. Fatigue.

[31] Levkulich N.C., Semiatin S.L., Gockel J.E., Middendorf J.R., DeWald A.T., Klingbeil N.W. (2019). The effect of process parameters on residual stress evolution and distortion in the laser powder bed fusion of Ti-6Al-4V. Addit. Manuf..

[32] Xing L.L., Zhang W.J., Zhao C.C., Gao W.Q., Shen Z.J., Liu W. (2021). Influence of powder bed temperature on the microstructure and mechanical properties of ti-6al-4v alloys fabricated via laser powder bed fusion. Materials.

[33] Pedrazzini S., Pek M.E., Ackerman A.K., Cheng Q., Ali H., Ghadbeigi H. (2023). Effect of Substrate Bed Temperature on Solute Segregation and Mechanical Properties in Ti-6Al-4V Produced by Laser Powder Bed Fusion. Metall. Mater. Trans. A.

[34] Shao J., Yu G., He X., Li S., Chen R., Zhao Y. (2019). Grain size evolution under different cooling rate in laser additive manufacturing of superalloy. Opt. Laser Technol..

[35] Scheel P., Markovic P., Van Petegem S., Makowska M.G., Wrobel R., Mayer T., Leinenbach C., Mazza E., Hosseini E. (2023). A close look at temperature profiles during laser powder bed fusion using operando X-ray diffraction and finite element simulations. Addit. Manuf. Lett..

[36] Zhang T., Liu C. (2022). Design of titanium alloys by additive manufacturing: A critical review. Adv. Powder Mater..

[37] Lakroune Y., Connétable D., Hugues J., Hermantier P., Barriobero-Vila P., Dehmas M. (2023). Microstructural evolution during post heat treatment of the Ti–6Al–4V alloy manufactured by laser powder bed fusion. J. Mater. Res. Technol..

[38] Mantri S.A., Banerjee R. (2018). Microstructure and micro-texture evolution of additively manufactured β-Ti alloys. Addit. Manuf..

[39] Shang G., Gan X., Wang X., Ge J., Li C., Zhu Z., Zhang X. (2024). Effect of Cooling Rate on α Variant Selection and Microstructure Evolution in TB17 Titanium Alloy. Mater. Artic..

[40] Derimow N., Benzing J.T., Joress H., Mcdannald A., Lu P., Delrio F.W., Moser N., Connolly M.J., Saville A.I., Kafka O.L. (2024). Microstructure and mechanical properties of laser powder bed fusion Ti-6Al-4V after HIP treatments with varied temperatures and cooling rates. Mater. Des..

[41] Karthikeyan T., Dasgupta A., Khatirkar R., Saroja S., Samajdar I., Vijayalakshmi M. (2010). Effect of cooling rate on transformation texture and variant selection during β→α transformation in Ti–5Ta–1.8Nb alloy. Mater. Sci. Eng. A.

[42] Agrawal A.K., Meric de Bellefon G., Thoma D. (2020). High-throughput experimentation for microstructural design in additively manufactured 316L stainless steel. Mater. Sci. Eng. A.

[43] Aboulkhair N.T., Simonelli M., Parry L., Ashcroft I., Tuck C., Hague R. (2019). 3D printing of Aluminium alloys: Additive Manufacturing of Aluminium alloys using selective laser melting. Prog. Mater. Sci..

[44] Elambasseril J., Rogers J., Wallbrink C., Munk D., Leary M., Qian M. (2023). Laser powder bed fusion additive manufacturing (LPBF-AM): The influence of design features and LPBF variables on surface topography and effect on fatigue properties. Crit. Rev. Solid State Mater. Sci..

[45] Gunenthiram V., Peyre P., Schneider M., Dal M., Coste F., Koutiri I., Fabbro R. (2018). Experimental analysis of spatter generation and melt-pool behavior during the powder bed laser beam melting process. J. Mater. Process. Technol..

[46] Scipioni Bertoli U., Wolfer A.J., Matthews M.J., Delplanque J.P.R., Schoenung J.M. (2017). On the limitations of Volumetric Energy Density as a design parameter for Selective Laser Melting. Mater. Des..

[47] Ahmad M., Farhana B., Foudzi M., Iliana F., Abu J., Sulong B. (2023). Review on volumetric energy density: Influence on morphology and mechanical properties of Ti6Al4V manufactured via laser powder bed fusion. Prog. Addit. Manuf..

[48] Wang H., Wang L., Cui R., Wang B., Luo L., Su Y. (2020). Differences in microstructure and nano-hardness of selective laser melted Inconel 718 single tracks under various melting modes of molten pool. J. Mater. Res. Technol..

[49] Carroll B.E., Palmer T.A., Beese A.M. (2015). Anisotropic tensile behavior of Ti-6Al-4V components fabricated with directed energy deposition additive manufacturing. Acta Mater..

[50] Prashanth K.G., Scudino S., Maity T., Das J., Eckert J. (2017). Is the energy density a reliable parameter for materials synthesis by selective laser melting?. Mater. Res. Lett..

[51] Niessen F., Nyysso T., Gazder A.A., Hielscher R. (2022). Parent grain reconstruction from partially or fully transformed microstructures in MTEX. J. Appl. Crystallogr..

[52] (2017). Metal-Methods for Estimating the Average Grain Size.

[53] (2013). Standard Test Method for Determining Residual Stresses by the Hole-Drilling Strain-Gage Method.

[54] Lu S.L., Todaro C.J., Sun Y.Y., Song T., Brandt M., Qian M. (2022). Variant selection in additively manufactured alpha-beta titanium alloys. J. Mater. Sci. Technol..

[55] Halder R., Sahoo S., Benzing J.T., Saville A.I., Rollett A.D. (2024). Variant selection and macrozone in Ti-6Al-4V walls during laser hot wire direct energy deposition. J. Mater. Res. Technol..

[56] Ahmed N., Barsoum I., Abu Al-Rub R.K. (2023). Numerical investigation of residual stresses in thin-walled additively manufactured structures from selective laser melting. Heliyon.

[57] Vrancken B., Cain V., Knutsen R., Van Humbeeck J. (2014). Residual stress via the contour method in compact tension specimens produced via selective laser melting. Scr. Mater..

[58] Aversa A., Carrozza A., Mercurio V., Calignano F., Sereda O., Pejchal V., Lombardi M. (2025). A Comparison Between the Residual Stresses of Ti6Al4V and Ti-6Al-2Sn-4Zr-6Mo Processed by Laser Powder Bed Fusion. Materials.

[59] Yin J., Yang L.L., Yang X., Zhu H., Wang D., Ke L., Wang Z., Wang G., Zeng X. (2019). High-power laser-matter interaction during laser powder bed fusion. Addit. Manuf..

[60] Montero-Sistiaga M.L., Pourbabak S., Van Humbeeck J., Schryvers D., Vanmeensel K. (2019). Microstructure and mechanical properties of Hastelloy X produced by HP-SLM (high power selective laser melting). Mater. Des..

[61] Guo W., Feng B., Yang Y., Ren Y., Liu Y., Yang H., Yang Q., Cui L., Tong X., Hao S. (2022). Effect of laser scanning speed on the microstructure, phase transformation and mechanical property of NiTi alloys fabricated by LPBF. Mater. Des..

[62] Louw D.F., Pistorius P.G.H. (2019). The effect of scan speed and hatch distance on prior-beta grain size in laser powder bed fused Ti-6Al-4V. Int. J. Adv. Manuf. Technol..

[63] Kosiba K., Gustmann T., Kim J.T., Seok J., Jung J., Beyer L., Scudino S., Giebeler L., Han J., Hufenbach J.K. (2023). Experimental cooling rates during high-power laser powder bed fusion at varying processing conditions. J. Alloys Compd..

[64] Chen Q., Xu L., Zhao L., Hao K., Han Y. (2023). Effect of scanning speed on microstructure and mechanical properties of as-printed Ti–22Al–25Nb intermetallic by laser powder bed fusion. Mater. Sci. Eng. A.

[65] Xxi I., Keller D., Wegener K., Stief P., Dantan J., Etienne A., Siadat A. (2022). Effect of Process Parameters on Melt Pool Geometry in Laser Powder Bed Fusion of Metals: A Numerical Investigation E ff ect of Process Parameters on Melt Pool Geometry in Laser Powder Bed. Procedia CIRP.

[66] Yang L., Ramachandran S., Bagasol A., Guan Q., Wang W., Browne D.J., Dowling D., Mirihanage W. (2023). Solidification microstructure variations in additively manufactured Ti-6Al-4V using laser powder bed fusion. Scr. Mater..

[67] Luo Q., Yin L., Simpson T.W., Beese A.M. (2022). Effect of processing parameters on pore structures, grain features, and mechanical properties in Ti-6Al-4V by laser powder bed fusion. Addit. Manuf..

[68] Dhiman S., Chinthapenta V., Brandt M., Fabijanic D., Xu W. (2024). Microstructure control in additively manufactured Ti-6Al-4V during high-power laser powder bed fusion. Addit. Manuf..

[69] Yang J., Yu H., Yin J., Gao M., Wang Z., Zeng X. (2016). Formation and control of martensite in Ti-6Al-4V alloy produced by selective laser melting. Mater. Des..

[70] Stanford N., Bate P.S. (2004). Crystallographic variant selection in Ti–6Al–4V. Acta Mater..

[71] Gao P., Fu M., Zhan M., Lei Z., Li Y. (2020). Deformation behavior and microstructure evolution of titanium alloys with lamellar microstructure in hot working process: A review. J. Mater. Sci. Technol..

[72] Xie Y., Gong M., Dong P., Zhou Q. (2025). Variant Selection in Laser-Powder Directed Energy Deposited Ti-6.5Al-3.5Mo-1.5Zr-0.3Si Titanium Alloy. Addit. Manuf. Front..

[73] Asherloo M., Wu Z., Sabisch J.E.C., Ghamarian I., Rollett A.D., Mostafaei A. (2023). Variant selection in laser powder b e d fusion of non-spherical Ti-6Al-4V powder. J. Mater. Sci. Technol..

[74] Yang C., Liu B., Pan L.L., Yang Y., Zhou Y., Cai W.S., Liu L. (2025). Disrupting variant selection memory effect in laser powder b e d fusion to improve strength-ductility synergy of Ti-6Al-4V alloys. J. Mater. Sci. Technol..

[75] Eliasu A., Hanson S., Kenneth D., Hukpati S., Yao M., Joseph A., Tetteh F., Czekanski A., Boakye S. (2022). Effect of individual printing parameters on residual stress and tribological behaviour of 316L stainless steel fabricated with laser powder bed fusion (L-PBF). Int. J. Adv. Manuf. Technol..

