# Supplementary material for: Microstructural Evolution and Mechanical Properties of LPBF Ti-6Al-4V with Different Process Parameters
Source: Materials (Basel). 2026 Mar 10;19(6):1049. doi: 10.3390/ma19061049 (PMC13027919; doi:10.3390/ma19061049)
Supplement: Supplementary file 1 [file materials-19-01049-s001.zip › Table S1.pdf]

| Laser power (W) | Scanning speed (mm/s) | Hatch distance (mm) |
|---|---|---|
| 173 | 900 | 0.08 |
| 173 | 1000 | 0.08 |
| 173 | 1100 | 0.08 |
| 173 | 900 | 0.06 |
| 173 | 1000 | 0.06 |
| 173 | 1100 | 0.06 |
| 173 | 1200 | 0.06 |
| 173 | 800 | 0.11 |
| 173 | 900 | 0.11 |
| 173 | 1000 | 0.11 |
| 173 | 1100 | 0.11 |
| 173 | 1200 | 0.11 |
| 173 | 664 | 0.11 |
| 173 | 900 | 0.14 |
| 173 | 1000 | 0.14 |
| 173 | 1100 | 0.14 |
| 173 | 664 | 0.14 |
| 173 | 800 | 0.16 |
| 183 | 900 | 0.08 |
| 183 | 1000 | 0.08 |
| 183 | 1100 | 0.08 |
| 183 | 1200 | 0.08 |
| 183 | 664 | 0.11 |
| 183 | 800 | 0.11 |
| 183 | 900 | 0.11 |
| 183 | 1100 | 0.11 |
| 183 | 1200 | 0.11 |
| 183 | 664 | 0.14 |
| 183 | 800 | 0.14 |
| 183 | 900 | 0.14 |
| 183 | 1000 | 0.14 |
| 183 | 1100 | 0.14 |
| 183 | 1200 | 0.14 |
| 183 | 664 | 0.16 |
| 183 | 800 | 0.16 |
| 183 | 900 | 0.16 |
| 183 | 1100 | 0.16 |
| 183 | 1200 | 0.06 |
| 193 | 664 | 0.11 |
| 193 | 800 | 0.11 |
| 193 | 900 | 0.11 |
| 193 | 1000 | 0.11 |
| 193 | 1100 | 0.11 |

| | | |
|---|---|---|
| 193 | 1200 | 0.11 |
| 193 | 664 | 0.14 |
| 193 | 900 | 0.14 |
| 193 | 1000 | 0.14 |
| 193 | 1100 | 0.14 |
| 173 | 800 | 0.08 |
| 193 | 800 | 0.08 |
| 173 | 1200 | 0.08 |
| 193 | 1200 | 0.08 |
| 173 | 800 | 0.14 |
| 193 | 800 | 0.14 |
| 173 | 1200 | 0.14 |
| 193 | 1200 | 0.14 |
| 183 | 664 | 0.11 |
