# Supplementary figures and images for: Microstructural Evolution and Mechanical Properties of LPBF Ti-6Al-4V with Different Process Parameters
Source: Materials (Basel). 2026 Mar 10;19(6):1049. doi: 10.3390/ma19061049 (PMC13027919; doi:10.3390/ma19061049)

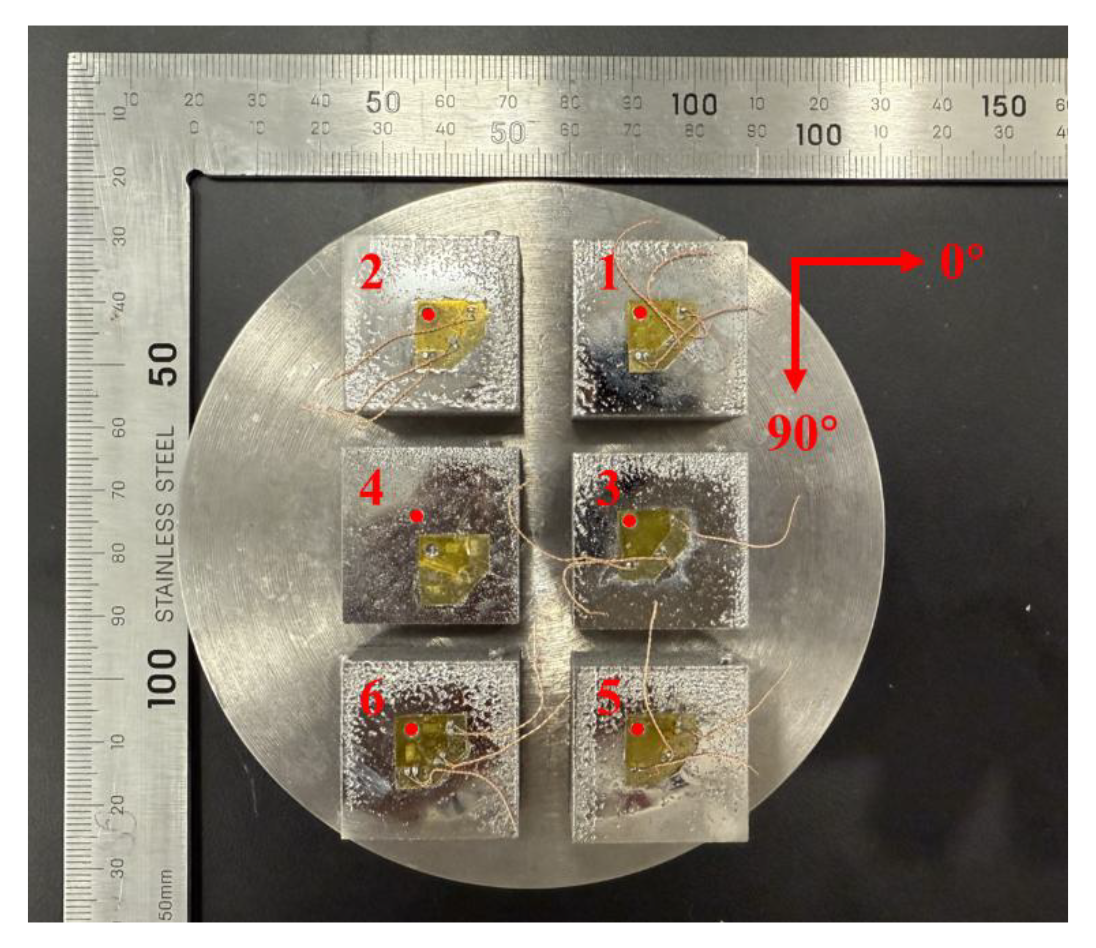

Supplement: Supplementary file 1 [file materials-19-01049-s001.zip › Figure S1.tif]
